# Exsolution of Fe-based pyramidal nanostructures from a noble metal doped perovskite matrix

**DOI:** 10.1039/d5na00469a

**Published:** 2025-08-29

**Authors:** Deblina Majumder, Shailza Saini, William S. J. Skinner, Alex Martinez Martin, Gwilherm Kerherve, David J. Payne, Debayan Mondal, Evangelos I. Papaioannou, Kalliopi Kousi

**Affiliations:** a School of Engineering, Newcastle University Newcastle upon Tyne NE1 7RU UK Deblina.Majumder@ncl.ac.uk; b School of Chemistry and Chemical Engineering, University of Surrey Surrey GU2 7XH UK; c Department of Materials, Imperial, College London London SW7 2AZ UK; d NEOM Education, Research, and Innovation Foundation Al Khuraybah Tabuk 49643-9136 Saudi Arabia; e Department of Condensed Matter and Materials Physics, S.N. Bose National Centre for Basic Sciences Kolkata 700 106 India

## Abstract

Homogeneous pyramidal oxide exsolution is demonstrated for the first time leveraging a La_0.05_Ag_0.05_Sr_0.90_FeO_3_ perovskite. The impressive structures were comprehensively characterised using XRD, SEM, TEM, XPS, and DFT to reveal their structural and chemical identity. The hierarchical oxide formation highlights the potential of exsolution as a nano-engineering method for developing oxide-based catalysts.

## Abstract 2

Fe-rich exsolved pyramidal nanostructures with reversible oxygen capacity acting as an oxygen reservoir.

## Graphical abstract

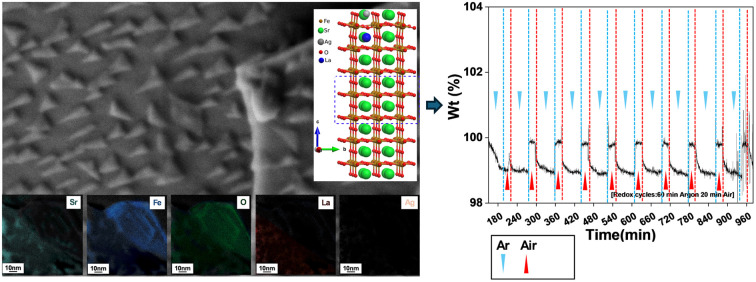

## Introduction

Transition metal oxides have long been recognised for their exceptional catalytic properties, with iron oxide standing out due to its abundance, cost-effectiveness, and remarkable performance in oxidation reactions, *i.e.* CO oxidation.^1,2^ The catalytic efficiency of iron oxide is intricately linked to its morphology, crystal structure, and surface characteristics, which influence the distribution of active sites towards adsorption of reactants and their subsequent conversion.^3^ Notably, the presence of Fe^2+^ and Fe^3+^ ions, along with their coordination environments, play a pivotal role towards the catalytic behaviour, with tetrahedrally coordinated Fe^3+^ centres exhibiting superior activity.^4,5^ Moreover, iron oxides seem to also be capable of forming diverse structures due to their ability to form various metal-to-oxygen ratio phases and stable oxidation states. The arrangement of iron ions within specific morphologies and reactive planes enhances oxygen mobility which directly impacts their catalytic efficacy,^6^ making them potential substitutes even for noble metal catalysts.^7^

Stimulated by the promising applications of iron oxide, various nanostructures have been synthesised as considerable research has revealed that the shape and exposed crystal planes of nanoparticles are crucial for appropriate adsorption, molecular trafficking, and desorption energies.^8,9^ Liu *et al.* reported iron oxide nanorods with reactive {110} crystal planes which due to the higher density of Fe atoms show superior catalytic activity compared to other morphologies.^10^ Similarly, Halim *et al.*^4^ investigated CO oxidation over nanocrystalline iron oxide highlighting the role of structural characteristics such as crystal size and shape in determining the activation energy and reaction kinetics.^11^ Iron oxide has been synthesised in various shapes, including nanowires, nanorods, nanoflowers, nanosheets, nanobelts, nanospheres, nanocubes, and nanocrystals, tailored for specific applications.^3^ However, conventional synthesis methods such as sol–gel, co-precipitation, and hydrothermal techniques often suffer from limitations like poor homogeneity and a tendency for particle sintering, which can compromise the uniformity of particle morphology and reduce active surface areas.^12^

Exsolution has revolutionised the field of heterogeneous catalysis by providing a controlled method for generating highly active and stable sites, overcoming the challenges such as sintering and detachment in conventional catalysts.^13^ This method involves controlled emergence of nanoparticles often from a perovskite host under reducing conditions, resulting in strong anchorage and uniform dispersion of active sites.^14^ By precisely controlling even trace amounts of dopants, exsolution facilitates the formation of regenerative catalysts with enhanced stability and catalytic performance under extreme conditions, such as high temperature and pressure.^15^ Depending on defect chemistry design, the active sites formed during exsolution can also dissolve back into the lattice upon oxidation and can be re-exsolved upon reduction, enabling regenerative catalytic activity.^16^ This methodology leverages the abundance and cost-effectiveness of transition metals.^17–19^ In particular, exsolution of Fe phases has proven to result in various morphologies, *i.e.*, whiskers or raspberry-like structures,^20–22^ of mixed metallic and oxide phases mostly owing to their Δ*G* value of the reduction.^18^ Recently, Zheng *et al.* reported the exsolution of Fe oxide towards the development of a robust FeO_*x*_/LaFeO_3_ heterostructure as a support, to construct a stable Pt-support interfaces achieving highly active CO oxidation at room temperature.^23^ While exsolution of Fe has been widely studied in electrochemical contexts,^20–22^ its oxide phase remains less explored despite its redox flexibility and catalytic versatility for processes like such as CO oxidation, Fischer–Tropsch synthesis, water splitting, and waste treatment.^23,24^

To the best of our knowledge, we report, for the first time, the controlled homogeneous formation of distinctive pyramidal iron oxide nanostructures from the La_0.05_Ag_0.05_Sr_0.9_FeO_3_ system *via* controlled exsolution. This study integrates X-ray diffraction, electron microscopy, and X-ray photoelectron spectroscopy (XPS) to gain unique elemental-level insights into the nature of these pyramidal nanostructures. The potential of this system has been evaluated in the context of reversible oxygen storage capacity, positioning it as a promising candidate for various catalytic applications. Furthermore, using density functional theory (DFT) analysis, we establish an *ab initio* thermodynamic framework that reveals the favourability of Fe oxide exsolution and the possible formation of sub-surface vacancies. Complementary molecular dynamics simulations provide additional insights, showing how the exsolution process is influenced by temperature and host-perovskite interactions.

## Results and discussion

**Fig. 1:**
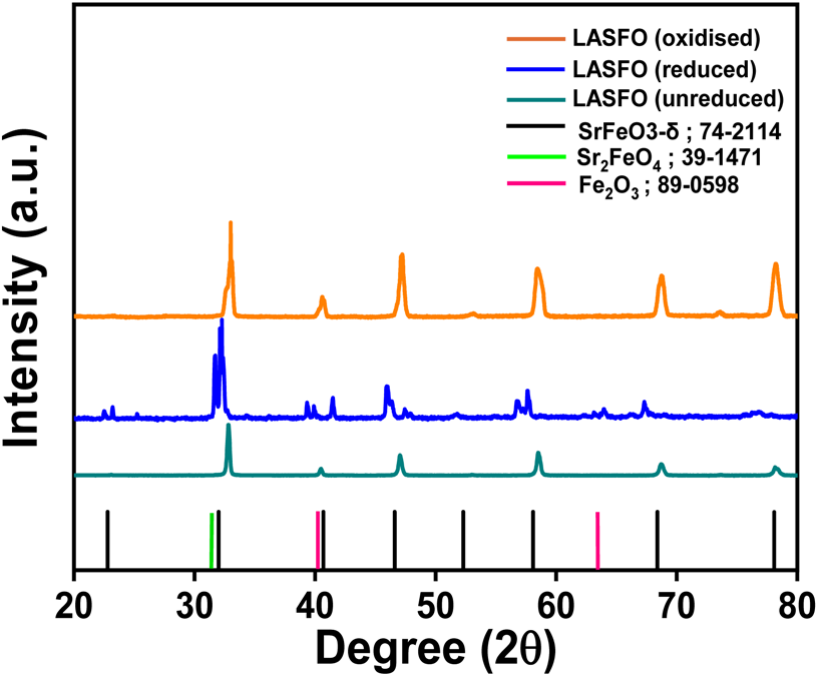
X-ray diffraction patterns of unreduced, reduced and oxidised LASFO.

We synthesized La_0.05_Ag_0.05_Sr_0.90_FeO_3_ (LASFO) *via* a solid-state method, doping the A-site of the perovskite structure with 0.05% La and Ag. The material was subsequently reduced in 5% H_2_ at 600 °C (details provided in the Methods and materials section) to provoke exsolution. To verify the intended composition, ICP-OES analysis confirmed the presence of La, Ag, Sr, and Fe (detailed in Table S1). Fig. 1 presents the XRD patterns of unreduced, reduced and oxidised LASFO. Reduced LASFO was subsequently re-oxidised to mimic the conditions of the redox applications for which these materials have been designed for. In the unreduced samples, the material exhibits a well-defined perovskite structure. Upon reduction, the perovskite undergoes a phase transition, forming a brownmillerite phase (Sr_2_Fe_2_O_5_), along with generation of an iron oxide (Fe_2_O_3_) phase^25^ (corresponding refinement in Fig. S1). Notably, upon oxidation, it reverts to formation the perovskite structure (oxidised), similar to the unreduced LASFO, although some morphological changes seem to be present indicated by the splitting of the peaks. This suggests the reversible nature of the material, indicating a flexible and dynamic host-lattice structure, likely driven by changes in the oxidation state of Fe, enabling the transition between perovskite and brownmillerite phases.

**Fig. 2:**
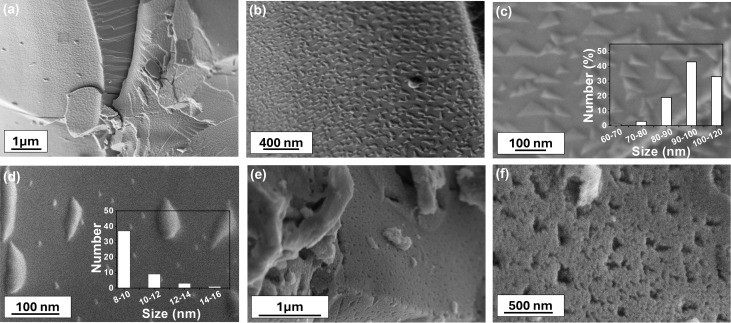
(a)–(c) SEM images of LASFO at different magnifications showing homogeneous formation of pyramidal structures where inset (c) shows their size distribution, (d) shows exsolved nanoparticles with their size distribution inset, (e) and (f) exhibits oxidised-LASFO forming pits on surface.

Fig. 2 presents SEM images of the reduced and oxidised LASFO at various magnifications, showcasing mainly the homogeneous exsolution of pyramidal structures and their dissolution after oxidation. These structures are uniformly distributed across the surface of the material, indicating a consistent and controlled synthesis process *via* exsolution. Few morphologies have been reported in the literature for exsolved Fe-based systems, including whisker-like and, FeO_*x*_ nanoparticles (formed *via* an “inside-out” mechanism on doped carbon nanoshells).^22,26^ Thalinger *et al.* observed the formation of uniform Fe and SrO rods, along with nanoparticles, upon the controlled reduction of La_0.6_Sr_0.4_FeO_3−*δ*_ (LSF) and Ni-LSF in different hydrogen environments.^26^ Nevertheless, even compared to these studies, the pyramidal exsolutions observed in our reduced LASFO samples exhibit a highly homogeneous distribution.^26^ The microscopic images (Fig. 2(a–c)) with varied magnification provide a detailed overview of this unique pyramidal phenomenon while Fig. 2(d) also indicates the additional formation of small round particles across the surface. The overall amount of the exsolved metal in the form of round nanoparticles is most likely too small to be detected by XRD or their peaks (iron metal) overlap with the peaks of the iron oxide phases (41° and 64°).^27^ The pyramidal structures on the other hand were distinctly visible due to their larger size and height, while the smaller nanoparticles, approximately one-tenth their size, were challenging to visualise due to disparities in the corresponding particle dimensions and focal depth and hence not seem to be prominent in appearance in Fig. 2(a)–(c). The size distribution of two distinct exsolved phases observed under SEM is analysed using ImageJ, with the insets of Fig. 2(c) and (d) showing pyramids and nanoparticles, respectively. Both the pyramidal structures and nanoparticles show a narrow size distribution. For the pyramidal structures, the most prevalent size range is 90–100 nm, comprising over 40% of the total distribution, while a smaller fraction falling within the 80–90 nm and 100–120 nm ranges. The nanoparticles on the other hand exhibit a smaller size distribution compared to the pyramids, with the dominant range being 8–10 nm, representing more than 40% of the total population while a minor fraction is distributed in the 11–12 nm range. Fig. 2(e) and (f) show that oxidation of the sample after exsolution resulted in the dissolution of the pyramidal structures back into the matrix, leaving behind corresponding pits at the surface.^28^ Re-reduction of the materials resulted in re-exsolution of some of the features (Fig. S3) although the mechanistic and chemistry of this process would need to be studied further in future research.

**Fig. 3:**
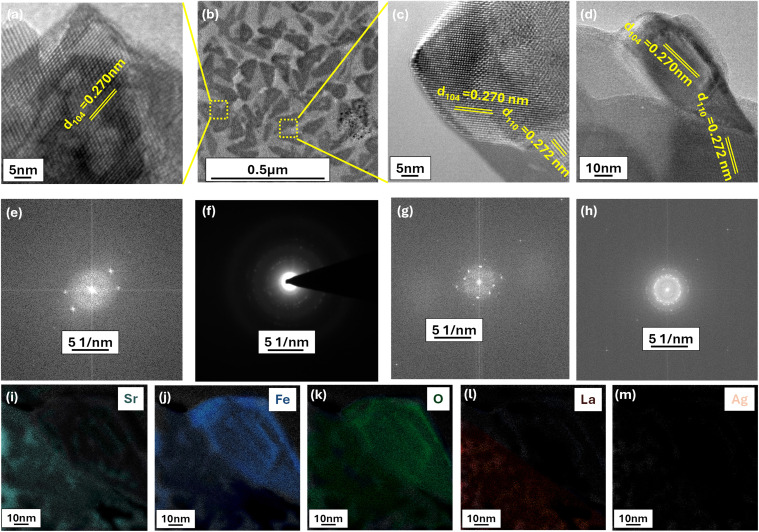
TEM analysis of LASFO. (a) and (c) presents HRTEM of selected magnified areas (with changed orientation) and highlighted in yellow (b); (d) shows HRTEM analysis of edge of a pyramid structure, (e); (g) and (h) include the live FFT pattern of the corresponding HRTEM analysis of (a), (c) and (d) respectively; (f) provides SAED pattern of (b); (i)–(m) represent the elemental analysis of pyramidal area of (d).

Elemental mapping *via* TEM and SEM was employed (Fig. 3 and S2) to probe the chemical composition of the emergent pyramid structures. Fig. 3(a) and (c) are magnified areas selected from Fig. 3(b) while Fig. 3(d) allows the clear deconvolution of the chemistry of our pyramids. Fig. 3(e), (g) and (h) represent the corresponding live FFT pattern of the HRTEM analysis shown in Fig. 3(a), (c) and 3(d) respectively. These HRTEM analysis in Fig. 3(a) and (c) shows characteristic lattice fringes (*d* spacing of 0.27 nm corresponding to 110 plane) of iron oxide, confirming their formation on the surface while *d*-spacing of 0.272 nm corroborates to surface of lanthanum strontium ferrite.^29–31^ Additionally, the selected area electron diffraction (SAED) pattern in Fig. 3(f) offers further confirmation of the crystalline phases present in the material.^32^ As evident in Fig. 3(i)–(m) the pyramidal structures appear to be primarily iron-based, however a critical observation of the Sr mapping in Fig. 3(i) suggests that Sr is also be present.

**Fig. 4:**
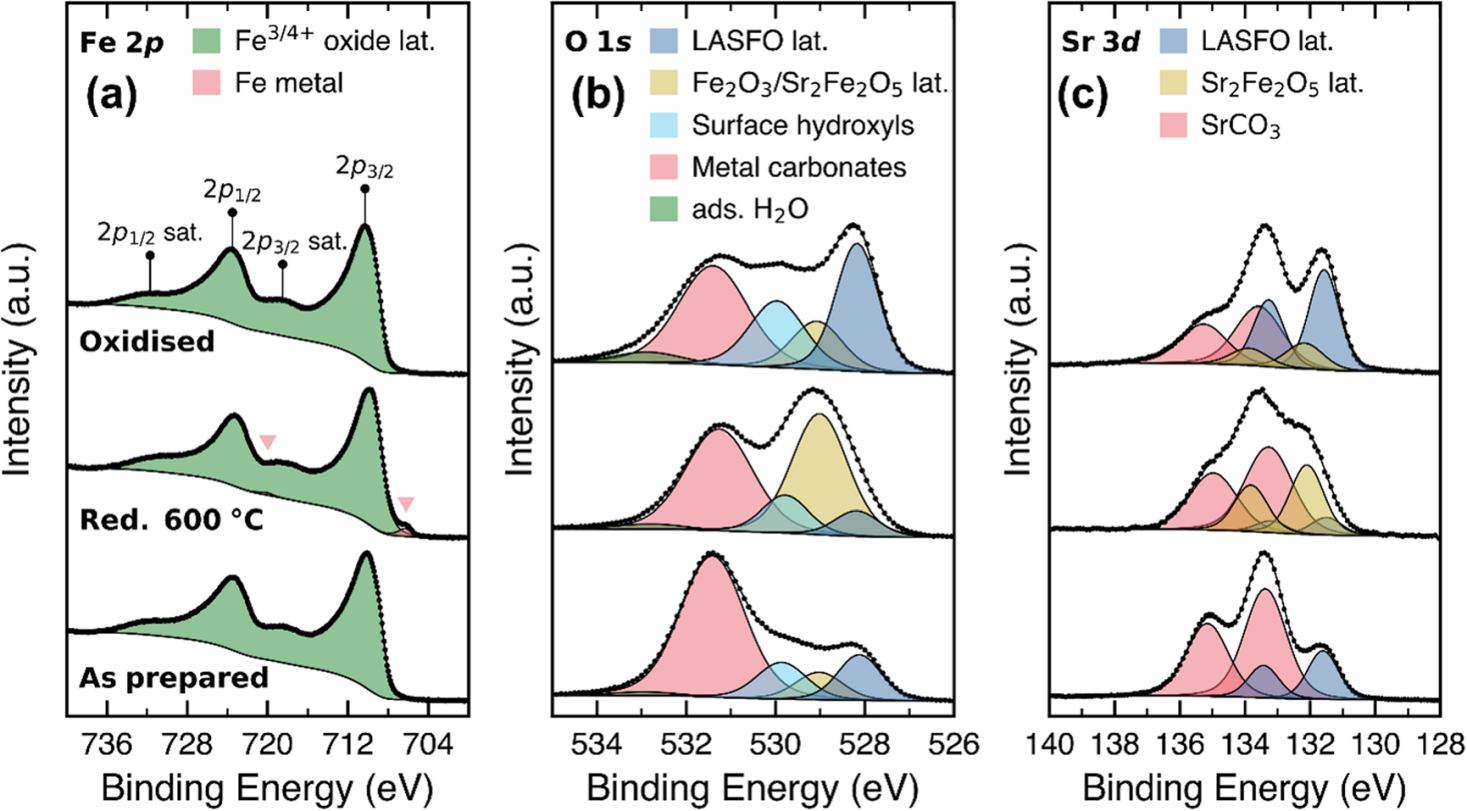
High-resolution core level scans of (a) Fe 2p, (b) O 1s and (c) Sr 3d. Measurements were performed on LASFO as prepared, after reduction (600 °C, 2 hours) and following subsequent oxidation. Core level spectra are normalised to the highest intensity in their spectrum to facilitate comparison between sample treatments.

XPS was performed to probe changes in surface chemistry and composition following reduction and subsequent oxidation. The Fe 2p, O 1s and Sr 3d core level spectra are plotted in Fig. 4, while the La 3d and C 1s core level spectra are plotted in Fig. S4. Compositional information derived from these fitted spectra is provided in Tables S2–S4.

The Fe 2p spectra of all samples in Fig. 4(a) exhibit a main Fe 2p_3/2_ peak centred close to 710 eV, and a characteristic shake-up satellite positioned approximately 8 eV higher, close to 718 eV. This closely resembles the complex spectral line shape that arises from both multiplet splitting of the core hole state and charge transfer shake-up processes typical of high-spin Fe^3+^ configurations.^33^ However, it is important to note that this does not preclude the presence of “Fe^4+^” – the formal oxidation state of Fe expected in A^2+^Fe^4+^O_3−*δ*_^2−^ compounds, the proportion of which will depend on the degree of oxygen non-stoichiometry. In placing quotation marks around Fe^4+^ here, we follow the convention of Rogge *et al.* to acknowledge that Fe is not strictly 3d^4^ (Fe^4+^) for intrinsic A^2+^Fe^4+^O_3_^2−^ compounds (in their case, CaFeO_3_); rather, Fe is understood to exist as a mixture of 3 d^4^ and 3d^5^L – strongly hybridized Fe^3+^, where an electron has been transferred from the O 2p orbital to the Fe 3d orbital, leaving behind a ligand hole in the O 2p orbital.^34–36^ In their work comparing the Fe 2p XPS spectra of LaFeO_3_ and CaFeO_3_ (with formal oxidation states of iron of +3 and +4, respectively), Rogge *et al.* note the challenges in differentiating between Fe^3+^ and “Fe^4+^” valence states, observing strikingly similar spectra for both compounds.^34^ This challenge in observing a distinct “Fe^4+^” valence state in the Fe 2p core level was also noted by Nenning *et al.*^37^ in their ambient pressure XPS study of La_0.6_Sr_0.4_FeO_3−*δ*_ and SrTi_0.7_Fe_0.3_O_3−*δ*_, who attributed the lack of direct spectroscopic evidence for “Fe^4+^” to either its absence from the surface (due to the ease of reduction at the oxide surface relative to the bulk), or to its XPS spectrum only weakly differing from that of Fe^3+^, as observed in the work of Rogge *et al.*^34^ Based on the findings of these works, we expect that the complex line shapes observed in the Fe 2p spectra in this work comprise contributions from varying proportions of the different Fe^3+^/“Fe^4+^” compounds identified with XRD – LASFO, Fe_2_O_3_ and Sr_2_Fe_2_O_5_. Interestingly, the Fe 2p spectrum retains a fairly consistent line shape following reduction and reoxidation, despite the phase transformations observed with XRD. The only slight difference between the spectra is an additional Fe 2p_3/2_ peak that appears close to 706 eV after reduction and disappears after subsequent oxidation. This peak is attributed to metallic Fe,^38^ which we anticipate is exsolved from the sample under reducing conditions, as observed in previous work on Ln_0.5_Ba_0.5_Fe_1−*x*_Ni_*x*_O_3_ (Ln = Pr, Sm; and *x* = 0, 0.1), and re-incorporated following oxidation.^39^ This may correspond to the small, round nanoparticles observed in the SEM images of the reduced sample.

The O 1s core level in Fig. 4(b) exhibits a complex line shape, which we have fitted with five peaks. Beginning on the low binding energy side, the peak centred close to 528 eV is attributed to oxygen in the perovskite lattice of LASFO, matching closely the position of the lattice oxygen peak previously observed for SrFeO_3−*δ*_.^40,41^ The intensity of this peak decreases slightly relative to the other peaks in the spectrum following reduction and significantly increases following subsequent oxidation. The next peak towards higher binding energies, centred close to 529 eV, demonstrates opposite behaviour to the peak attributed to LASFO – a significant relative increase following reduction, followed by a decrease after re-oxidation. Based on this behaviour, we tentatively propose this peak to comprise contributions from both Fe_2_O_3_ and Sr_2_Fe_2_O_5_ – the two phases observed with XRD following reduction. Literature O 1s binding energies for Fe_2_O_3_ show some degree of variation, but typically lie within the 529.7–529.9 eV range.^42–45^ Few XPS studies of Sr_2_Fe_2_O_5_ have been performed, but literature O 1s binding energies of similar Ca_2_Fe_2_O_5_ compounds lie in the 528.7–529.4 range.^46–48^ The Fe 2p–O 1s separation of various Fe^3+^ compounds including Fe_2_O_3_ has been found to lie in the 180.7–181.0 eV range^49^ which matches very closely to the separation of approximately 181 eV between the main Fe 2p_3/2_ peak and the O 1s peak attributed to Fe_2_O_3_ and Sr_2_Fe_2_O_5_ in this work. Therefore, a single broad line shape was fitted to encompass contributions from both Fe_2_O_3_ and Sr_2_Fe_2_O_5_ phases, rather than attempting to fit individual peaks for both species. The separation of 1 eV between this peak and the lower binding energy peak attributed to LASFO may be related to the effects of screening conduction electrons present in metallic SrFeO_3−*δ*_^49^ which are absent in Fe_2_O_3_ and Sr_2_Fe_2_O_5_. More efficient final-state screening by conduction electrons in metallic SrFeO_3−*δ*_ would be expected to lower the core hole energy, thereby reducing the measured O 1s binding energy. The remaining three peaks to the high binding energy side of the O 1s core level spectrum are attributed to surface hydroxyls, metal carbonates and adsorbed water, respectively, based on their relative separations from the bulk lattice oxide peak.^50,51^ Some slight changes in the relative proportions of these peaks are observed between treatments, but there is no notable trend following reduction and re-oxidation.

The Sr 3d core level spectra in Fig. 4(d) also exhibit a complex line shape, which has been fitted with three doublets. The lowest binding energy doublet (with Sr 3d_5/2_ (ref. 46–48) centred close to 131.6 eV) is attributed to strontium in the LASFO perovskite lattice, exhibiting a similar binding energy to that observed in previous XPS measurements of SrFeO_3−*δ*_.^40^ The relative intensity of this doublet decreases slightly following reduction, and increases relative to the other peaks in the spectrum following subsequent oxidation, demonstrating a very similar trend to that observed for the lowest binding energy peak in the O 1s spectrum, which was also attributed to LASFO. The next doublet towards higher binding energies (with Sr 3d_5/2_ centred close to 132.1 eV) emerges following reduction and mostly disappears following subsequent oxidation. This doublet exhibits a very similar trend to that observed for the second lowest binding energy peak in the O 1s core level and is therefore attributed to the Sr_2_Fe_2_O_5_ phase formed following reduction. Similar to the LASFO and Fe_2_O_3_/Sr_2_Fe_2_O_5_ peaks in the O 1s core level, we anticipate that the lower binding energy position of LASFO compared to that of Sr_2_Fe_2_O_5_ in the Sr 3d core level can also be attributed to the enhanced final state screening effects in metallic SrFeO_3_. The highest binding energy doublet (with Sr 3d_5/2_ centred at 133.4 eV) is attributed to SrCO_3_, demonstrating a similar binding energy position to that observed in previous measurements of pure SrCO_3_ (ref. 52) and perovskite compounds where SrCO_3_ has been formed at the surface.^53^

The La 3d_5/2_ spectra in Fig. S4(a) exhibit broad main and satellite peaks, centred close to 834.4 and 837.8 eV, respectively, with a corresponding separation of approximately 3.4 eV. The breadth of the La 3d_5/2_ main line and satellite, and their overlap between them, makes extraction of an accurate peak position and main-satellite separation challenging; however, it is clear that the approximate separation of 3.4 eV is much lower than that observed for La_2_O_3_ (4.3–4.9)^54–57^ and closer to those observed for La(OH)_3_ (3.7–3.9)^55,57^ and La_2_(CO_3_)_3_ (3.5–3.8). However, the binding energy position of the La 3d_5/2_ main line at approximately 834.4 eV lies closer to those of La_2_O_3_ (834.8–834.9 eV)^56,57^ and La(OH)_3_ (835.1 eV)^57^ than that of La_2_(CO_3_)_3_ (835.6 eV).^58^ This suggests that La^3+^ in the surface region may exist in a complex mixture of oxide, hydroxide and carbonate states. The consistent separation between main and satellite peak following reduction and oxidation suggests that these states remain relatively unchanged following the sample treatments employed.

## Density functional theory analysis

**Fig. 5:**
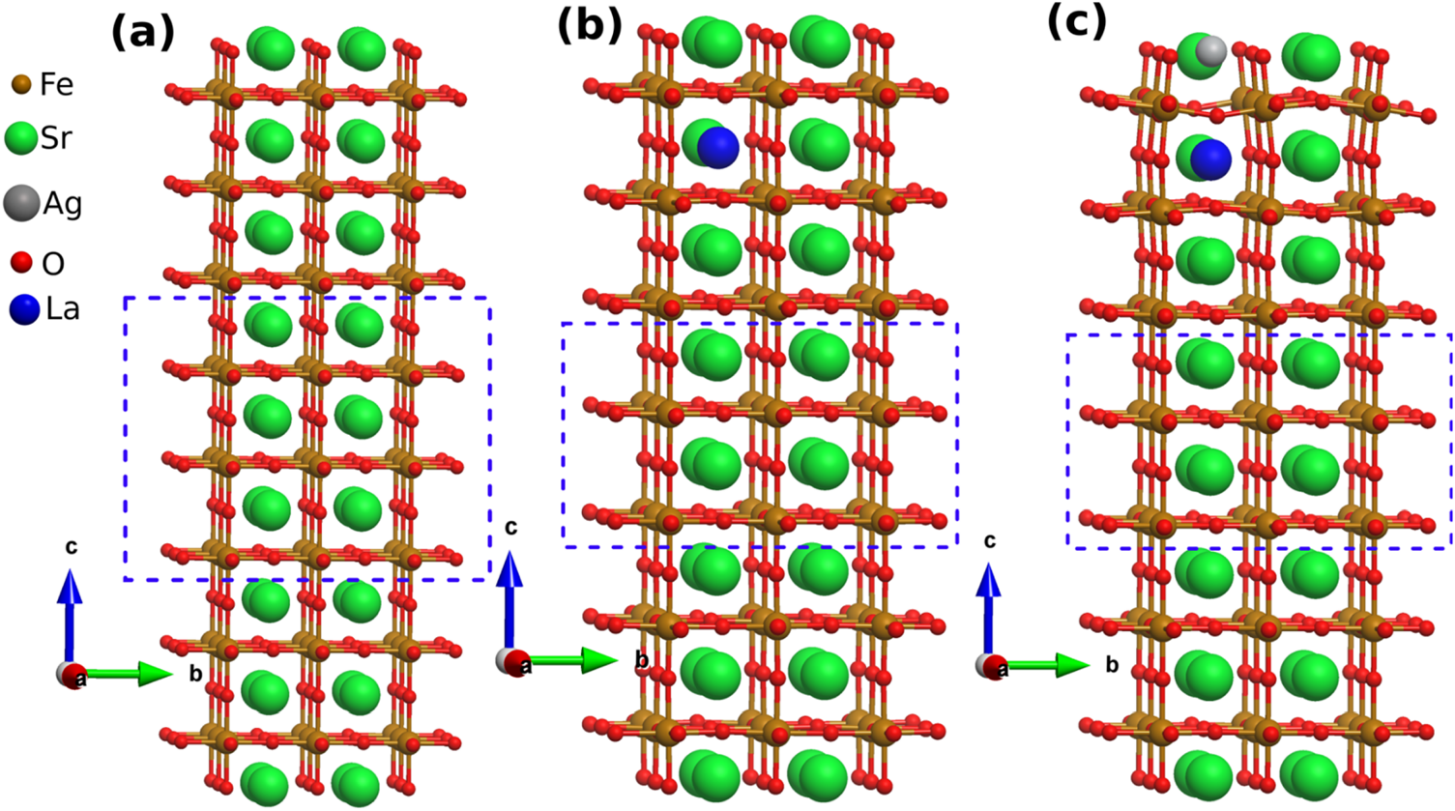
Theoretical model of (a) pristine SFO (b) LSFO and (c) LASFO. The blue box denotes the fixed layers simulated as bulk structure.

As no pyramids were detected in samples without Ag doping (Fig. S5), we gathered that the contribution of Ag could be the determining factor for the formation of such structures, hence we investigated the contribution of Ag towards the exsolution of our pyramidal structures using DFT analysis. We deployed Strontium ferrite perovskite system (SFO), and La doped SFO with and without Ag and are termed as LASFO and LSFO respectively as presented in Fig. 5(a)–(c).

**Fig. 6:**
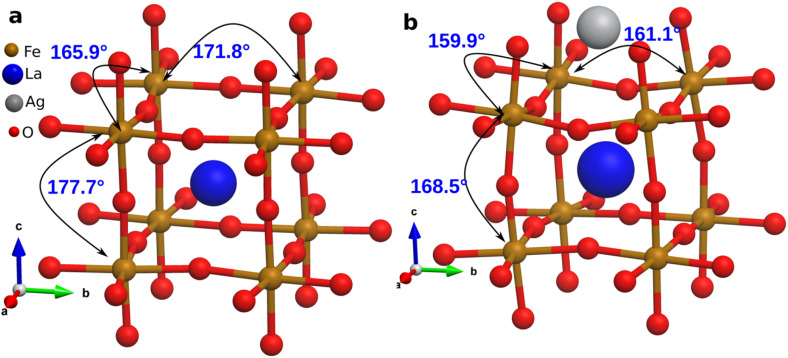
Fe–O–Fe bond angles of relaxed LSFO (a) before and (b) after Ag is introduced.

LASFO is a mixed-valence perovskite oxide where iron ions can exist in multiple oxidation states, typically Fe^3+^ (3d^5^, high-spin S = 5/2) and Fe^2+^ (3d^4^, high-spin or low-spin).^59^ To verify the presence of Fe^4+^ in undoped LSFO, we calculated the Bader charge of Fe ions and observed an average value of +2.4*e*, consistent with a mixture of Fe^3+^ and Fe^4+^ states.^60–62^ This aligns with experimental studies using XPS, which confirms the presence of Fe^4+^ is due to lattice-induced strain and oxygen anion coordination. After Ag doping, this value decreases to +2.1*e*, reflecting an enhanced stabilisation of Fe^3+^ as Fe^3+^ typically has a lower charge state (closer to +3) compared to Fe^4+^. Due to Ag doping, the Fe–O–Fe bond angles decrease (Fig. 6) from an average of 171.83° in LSFO to 163.17° in LASFO. In undoped LSFO, the average Fe–O–Fe bond angle along the *x*-crystal axis is 165.9°, whereas in the Ag-doped structure, this angle is further reduced to 159.5° (Fig. 6). The corresponding structural distortions are summarised in Table 1. This systematic reduction in bond angles enhances the overlap between Fe 3d orbitals and O 2p orbitals, promoting superexchange interactions that stabilise the Fe^3+^ oxidation state (see SI for detailed super-exchange mechanism analysis).^53,63–70^

**Table 1:** Fe–O–Fe bond angles in LSFO and LASFO

Structure	Bond angle 1 (°)	Bond angle 1 (°)	Bond angle 1 (°)	Average bond angle 1 (°)
LSFO	171.85	165.95	177.70	171.83
LASFO	159.93	161.09	168.50	163.17

A stabilised Fe^3+^ state influences the oxygen vacancy formation energy, potentially making it easier for oxygen vacancies to form, as Fe^3+^ ions can better accommodate the reduced charge density and compensate for the charge imbalance created by the missing oxygen atoms. Upon the formation of oxygen vacancies, Fe^3+^ undergoes partial reduction to Fe^2+^ to compensate for the charge imbalance caused by the missing oxygen atom (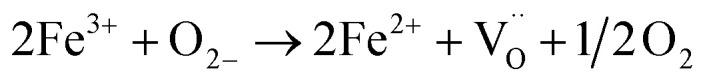; where 
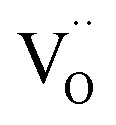
 represents a doubly charged oxygen vacancy). During reduction, Fe exsolution is closely tied to oxygen vacancy formation and the progressive reduction of Fe ions through the stepwise mechanism: formation of oxygen vacancies and Fe^2+^, followed by further reduction to metallic Fe (Fe^2+^ + 2e^−^ → Fe^0^) *via* successive electron transfers under reducing conditions. The energetic analysis of oxygen vacancy formation before and after incorporating Ag in LSFO reveals the critical role of dopants in modulating oxygen vacancy stability. When Ag is introduced into the A-site of LSFO (partially replacing La or Sr), it significantly alters the system behaviour due to its distinct valence state and ionic radius. Ag^+^ ions, being more polarisable, interact differently with the oxygen sublattice, modifying the local electrostatic environment and weakening some Fe–O bonds, which facilitates oxygen removal under reducing conditions. This results in an oxygen vacancy formation energy of −2.23 eV, which is substantially lower than that of LSFO (−0.246 eV), making vacancy formation more favourable in the Ag-doped system. This localised weakening of Fe–O bonds and increased oxygen mobility drive the transition to the brownmillerite structure, which naturally accommodates a higher density of oxygen vacancies. Thus, Ag incorporation not only enhances the formation of oxygen vacancies but also accelerates the structural transformation to the brownmillerite phase.

## Oxygen capacity and redox behaviour

**Fig. 7:**
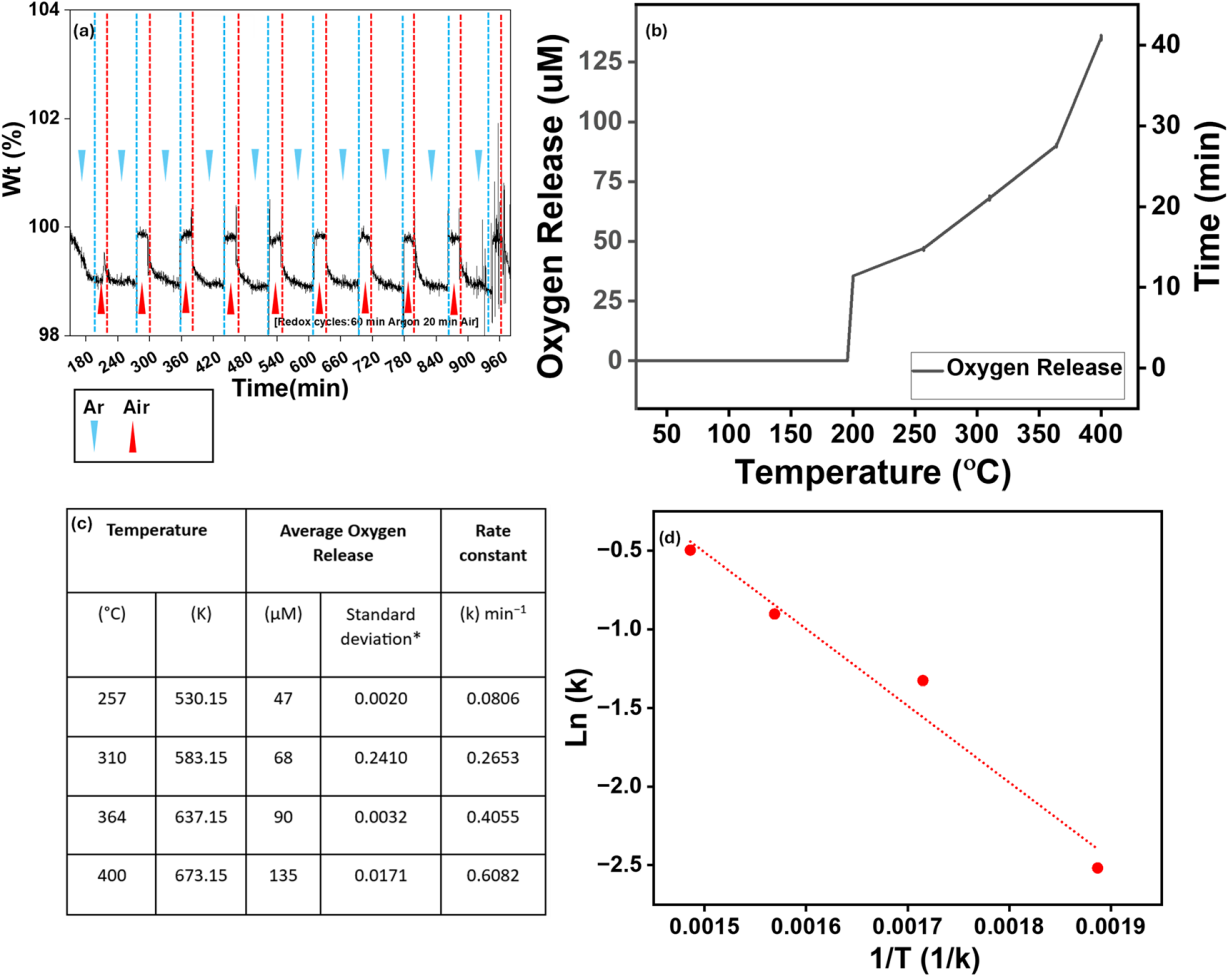
(a) Isothermal gas cycling over LASFO at 400 °C where vertical lines indicate switching between Ar and air (20% oxygen) during thermogravimetric analysis, (b) TPD of LASFO under 5% He, (c) and (d) corresponding average oxygen release parameters (in chart) and (d) Arrhenius plot of TPD analysis respectively.

The thermogravimetric analysis (TGA) was conducted on LASFO samples to evaluate their reversible oxygen capacity and the reversible nature of oxygen release during redox cycling. The system was subjected to a series of 60-minute reduction under Argon and 20-minute oxidation in air at a flow rate of 200 mL min^−1^ at 400 °C. The average weight loss during each redox cycle was found to be 1.04% for LASFO indicating that the oxygen capacity of 0.063 mol O_2_ per mol. The rate of oxygen release was analysed by examining the fourth cycle of the TGA curves in Fig. 7, and the corresponding morphology of LASFO is included in Fig. S6(a), showing structural integrity and indicating thermal stability under repeated cycles at high temperature.

The slopes of the reduction phases were calculated to determine the rate of oxygen release, and the oxidation potential is calculated from the weight gain during the oxidation phase. This shows oxygen release potential of LASFO, making it a suitable candidate for applications requiring efficient oxygen exchange. Temperature programmed desorption (TPD) analysis was conducted on exsolved LASFO under a 5% He atmosphere to investigate the intrinsic oxygen release behaviour without any prior adsorption. Unlike conventional TPD studies, which typically involve desorption of pre-adsorbed molecules, this study focuses on the inherent oxygen evolution from the material itself. As shown in Fig. 7(b), oxygen release starts at approximately 200 °C. The time parameter is introduced to determine the associated rate constant of oxygen release, as it is calculated based on the time elapsed from the onset of oxygen evolution. The primary oxygen release is observed at ∼250 °C, and further temperature increments of 50 °C were applied only after saturation was reached to track progressive oxygen evolution accurately. The corresponding parameters are listed in Fig. 7(c) and (d)^71–73^ shows the Arrhenius plot with linear regression and the activation energy for the oxygen desorption process (assuming first-order kinetics) is ∼40.59 kJ mol^−1^.

This provides critical insights into the oxygen desorption kinetics of LASFO as its low activation energy indicates efficient and reversible oxygen release at temperatures (∼250–400 °C), which is a key advantage over conventional perovskite oxides that typically require much higher temperatures for oxygen exchange as reported in the literature.^74,75^ To evaluate the catalytic performance of LASFO in comparison with traditional catalyst systems such as Pt-based catalysts supported on conventional oxides like Al_2_O_3_ and CeO_2_, CO oxidation was chosen as a representative model reaction.^76–79^ Under standard fixed-bed quartz reactor flow conditions (20 mg of catalyst exposed to 0.6% CO and 1 kPa O_2_ in a total flow of 150 mL min^−1^ at normal temperature and pressure) LASFO exhibited a CO conversion onset above 180 °C (Fig. S6b). Given that conversion efficiency can be improved by varying reaction conditions, the critical finding is the demonstrable catalytic activity of LASFO towards CO oxidation.

Our study demonstrates the exsolution of uniform and controlled pyramidal nanostructures with enhanced oxygen storage capacity. However, further analysis is required to gain deeper insights into the mechanisms governing their formation and to evaluate their potential significance in catalysis, particularly in relation to their stability, reactivity, and regeneration under operational conditions.

## Conclusion

This study presents a novel approach to material design, showcasing the simultaneous exsolution of metal and metal oxide phases and the formation of uniquely structured pyramidal nanostructures *via* exsolution. Through the acquired doping strategy involving trace amounts of noble metals, it elucidates how subtle A-site modifications in perovskites influence bond angles, enable the homogeneous emergence of unique morphologies. Advanced characterisation techniques, including electron microscopy and XPS, have been pivotal in investigating the elemental composition and structural features of these hierarchical nanostructures, confirming their exquisite morphology and composition. The reversible oxygen release and storage capacity of these structures at low temperatures mark them as a versatile platform for advanced catalytic applications. Furthermore, the study introduces a first-of-its-kind methodology in exsolution research, opening a new horizon in heterogeneous catalysis. The anchored and reversible nature of the iron-based exsolved oxide offer active sites towards exceptional cyclic redox stability. This synergy of experimental and theoretical insights into metal oxide exsolution paves the way for designing hierarchical structures of various metal oxides, unlocking significant potential for robust *in situ* catalytic applications across diverse domains.

## Materials and method

### Material synthesis

Perovskite oxides were synthesized *via* a modified solid-state reaction. High-purity precursors, including lanthanum nitrate hexahydrate (La(NO_3_)_3_·6H_2_O), strontium carbonate (SrCO_3_), silver nitrate (AgNO_3_), and ferric nitrate nonahydrate (Fe(NO_3_)_3_·9H_2_O), were combined in stoichiometric ratios. The mixture was quantitatively transferred to a beaker and mixed with acetone and 0.05 wt% Hypermer KD1 dispersant. To ensure homogeneity and break down any agglomerates, an ultrasonic Hielscher UP200S probe was employed, resulting in a fine and stable dispersion. The acetone was then evaporated by maintaining the mixture at 75 °C overnight in an oven. The dried mixture was transferred to an alumina crucible and calcined at 1000 °C for 6 hours to decompose the carbonates and initiate the formation of the perovskite phase. The resulting calcined powder was pressed into pellets and sintered at 1400 °C for 15 hours to achieve complete formation of the perovskite phase. These perovskite pellets were subsequently crushed and sieved to obtain powders with particle sizes ranging from 80 to 160 μm. The exsolution process was conducted by surface reduction in a controlled atmosphere furnace. The perovskite powders were subjected to a continuous flow of 5% H_2_/Ar at 600 °C for 2 hours, with a cooling rate of 10 °C min^−1^ to facilitate the exsolution process.

### Materials characterisation

XRD was used to measure crystallinity of the materials, using a PANalaytical X'Pert Pro, coupled to a X'Celerator detector. Used radiation was Cu Kα (*λ* = 1.5409 Å; 40 kV voltage). Scans were done over the range 20–90° in 2*θ*, step size of 0.0167°, 8 s per step. A 2° anti-scatter slit and 0.02° fixed divergence slits were used together with a beam mask of 20 mm. Refinement of the PXRD spectra was conducted in GSAS-II. For the as sintered catalysts, the starting model was Pm-3m (SrFeO_3_, ICSD code 91062), and cubic Ag *Fm*3̄*m* (Ag, ICSD code 22434). Occupancy was kept as 1 for the A-site atoms. Background, cell parameters, and scale were refined. For the reduced materials, a tetragonal model was used, *I*4/*mmm* (SrFeO_2.875_, ICSD code 8618 and Sr_2_FeO_4_, ICSD code 74419), and *R*3̄*c* (Fe_2_O_3_, ICSD code 7797).

Scanning electron microscopy (SEM) was carried out using a Thermo Fisher Apreo 2 scanning SEM and TESCAN MIRA 3 FEG-SEM, equipped with a field-emission gun for high-resolution imaging, to examine surface features, particle distribution, and hierarchical structures with precise spatial resolution. For the pyramidal structures, the hypotenuse length was considered as it is the most distinguishable and consistent dimension observable in 2D SEM images, correlating with particle size and geometric properties like surface area and volume. This measurement ensures accuracy and ease of comparison, as features like base width cannot be precisely determined due to projection effects, where the three-dimensional morphology is compressed into a two-dimensional image, leading to distortions in perceived dimensions and obscuring true structural proportions. Capturing nanoparticles required reducing the working distance (WD) at 1 mm and tilting the sample stage, which improved resolution and provided clarity for fine features. For surface feature analysis, WD was secured at 0.7 mm with view field 1 μm. Tilting the sample stage further improves visibility by altering the observation angle, enhancing depth perception, and capturing otherwise obscured features. However, this tilting introduced slight distortions, causing adjacent pyramidal structures to appear elongated due to changes in perspective and projection effects. ImageJ software was employed to analyse the acquired images for particle size distribution and the population of the exsolved particles.

TGA was performed using a Rubotherm dynTHERM unit to investigate the redox behaviour of the samples. A weighted amount of 200 mg of the sample was placed in the TGA crucible, and the gas flow rate was maintained at 200 mL min^−1^. The sample was heated to 400 °C at a rate of 10 °C min^−1^ under atmospheric pressure. To assess redox cycling stability, the sample underwent a series of alternating reduction and oxidation cycles, consisting of 60-minute reduction under argon Ar followed by 20-minute oxidation in air, both conducted at 400 °C and a constant flow rate of 200 mL min^−1^. The mass changes were continuously recorded to monitor the oxygen exchange capacity during cycling.

TPD experiments were conducted in a continuous-flow, single-chamber reactor at atmospheric pressure by introducing 100 mg of sample. The sample temperature was monitored using a K-type thermocouple, positioned in proximity to the catalyst surface. The gas flow was regulated using digitally interfaced mass flow controllers, with a constant flow of 5% He at 50 mL min^−1^, supplied by BOC Ltd. The reactor temperature was ramped from room temperature to 400 °C at a heating rate of 10 °C min^−1^ under a continuous flow of He at atmospheric pressure. The desorption of oxygen was continuously monitored using a Hiden Analytical mass spectrometer (HAS-301-1291). To determine Arrhenius plot for TPD analysis, the following eqn (1) is used

1 *k* = *A*e_*RT*_^−*E*_a_^

where, *k* is the rate constant, *A* is the pre-exponential factor (2.5 × 104 s^−1^), *E*_a_ is the activation energy, *R* is the universal gas constant (8.314 J mol^−1^ K^−1^) and *T* is the temperature in Kelvin.^80^

Under the similar set up, approximately 20 mg of catalyst was placed on quartz wool in a vertical, fixed-bed reactor (30 cm long, 1 cm inner diameter). Gas flow, controlled by electronic mass flow controllers, was fed from the top, and a K-type thermocouple monitored the temperature near the catalytic bed. The gases used were high purity 20% CO/He, 20% O_2_/He, and CP grade He (N5, 99.999% purity) from BOC Ltd. The total gas flow rate was kept constant at 1 × 10^−4^ mol s^−1^ m^−2^ (150 cm^3^ min^−1^) at normal temperature and pressure (NTP), measured at the outlet using a Varian digital flow meter (1000 series). The production rate of CO_2_ (*r*CO_2_) in mol (CO_2_) per s is calculated as: *r*_CO_2__ = *y*_CO_2__ × *n*; where *y*_CO_2__ is the measured CO_2_ mole fraction at the gas outlet and *n* is the molar flow rate.

X-ray photoelectron spectroscopy (XPS) spectra were recorded using a K-Alpha^+^ spectrometer (Thermo Scientific), which operates at a base pressure of 8 × 10^−9^ mbar and incorporates a monochromated, micro-focused Al Kα X-ray source (*E* = 1486.6 eV) and a 180° double focusing hemispherical analyser. The X-ray source was operated at 12 kV anode bias and 6 mA emission current, and the maximum spot size was 400 μm^2^. Sample charging was minimised using an in-built dual-beam source combining co-axial ultra-low energy electron and Ar^+^ ion beams. Powder samples were mounted onto a tantalum sample plate with conductive copper tape. Core-level and survey spectra were recorded at pass energies of 20 and 200 eV, respectively. Peak fitting was performed using the Thermo Avantage software; a Shirley background was applied, and the shape of all peaks was constrained to be a convolution of Gaussian and Lorentzian line shapes (80% Gaussian-20% Lorentzian, with no asymmetry). Binding energy correction was performed by setting the C 1s peak of adventitious carbon (C–C) at 284.8 eV, with all other peaks shifted accordingly. An estimate for the elemental composition of each sample (Tables S.2–4) was obtained with the Thermo Avantage software, using the areas of the relevant peaks (corrected with the TPP-2M approach) and their respective sensitivity factors.

All structural optimisations and static calculations were performed using spin-polarized density functional theory (DFT), as implemented in the Vienna *Ab initio* Simulation Package (VASP)^81,82^ with the projector augmented wave (PAW) method.^83,84^ The generalised gradient approximation (GGA) functional of Perdew, Burke, and Ernzerhof (PBE)^85^ was used to describe the exchange-correlation potential. The electronic configurations of La: 4f, 5d, and 6s states, Fe: 3d and 4s states O: 2s and 2p states treated as valence states. Since standard GGA-DFT underestimates the localization effects of Fe 3d orbitals, the Hubbard-*U* approach (DFT + *U*) was employed to improve the accuracy of electronic structure calculations. The effective Hubbard *U* parameter (*U*_eff_ = *U* − *J*) was incorporated following the approach of Dudarev *et al.*,^86^ where only the *U* term was explicitly considered. For Fe atoms, we used a *U*_eff_ of 4.0 eV, consistent with previous studies on iron-based perovskites. This value is within the range of *U* values (2–4 eV) reported in literature,^87,88^ chosen based on comparisons with experimental magnetic moments and structural parameters. Other studies have utilised different *U* values depending on the oxidation states of Fe; for example, Mosey *et al.*^89^ applied *U*_eff_ = 3.7 eV for Fe^2+^ and 4.3 eV for Fe^3+^, yielding accurate lattice parameters and band gaps for FeO and Fe_2_O_3_, respectively. However, for systems containing mixed-valent Fe (Fe^3+^/Fe^4+^), such as LSFO, a single *U* parameter was required to maintain consistency across different oxidation states.

For surface calculations, we constructed an SrFeO_3_ (SFO) slab model with 8 alternating SrO and FeO_2_22_ layers, maintaining stoichiometry. Among possible surface terminations, the SrO-terminated slab was found to be the most energetically stable and was chosen for further investigation. The final model consists of 36 Sr, 32 Fe, and 100 O atoms in a periodic slab geometry. A minimum vacuum spacing of 12 Å was introduced to eliminate interactions between periodic images. To simulate La doping, two next-nearest-neighbour Sr atoms were replaced with La, corresponding to a composition of La_0.05_Sr_0.95_FeO_3_(LSFO). Similarly, Ag doping was modelled by substituting Sr with Ag at different lattice positions in both the bulk and surface regions to assess the stability and incorporation preference of Ag dopants. All structures were optimized until the maximum force on any atom was less than 0.03 eV Å^−1^. The Brillouin zone was sampled using a 5 × 5 × 1 Monkhorst–Pack grid, ensuring convergence of total energies and electronic properties. The bottom two atomic layers of the slab were fixed to their bulk lattice positions to mimic the semi-infinite nature of the surface, while all other layers were fully relaxed.

Bader charge analysis was performed to estimate the oxidation states of Fe ions in the materials. This analysis technique, developed by Sanville *et al.*,^90^ partitions the charge density based on zero-flux surfaces in the charge density gradient.^91^ The approach works by first identifying local maxima in the electron density distribution then partitioning the total charge density into regions bounded by surfaces where the gradient of the charge density is zero perpendicular to the surface (zero-flux surfaces) and finally assigning the total charge within each region to the atom located at the corresponding local maximum. The Bader method provides a physically meaningful way to partition the total charge density and estimate the oxidation states of atoms in a material. This is particularly useful for complex systems where formal oxidation states may not be straightforward to determine. The relationship between Bader charges and oxidation states is not direct, but comparative analysis with reference systems can provide reliable estimates. The Bader charge values are typically lower than the formal oxidation states due to the continuous nature of the electron density and the incomplete electron transfer in chemical bonds with partial ionic character.

## Conflicts of interest

There are no conflicts to declare.

## Supplementary Material

NA-OLF-D5NA00469A-s001

NA-OLF-D5NA00469A-s002

NA-OLF-D5NA00469A-s003

NA-OLF-D5NA00469A-s004

NA-OLF-D5NA00469A-s005

NA-OLF-D5NA00469A-s006

NA-OLF-D5NA00469A-s007

NA-OLF-D5NA00469A-s008

## Data Availability

The research data for this article can be accessed at DOI: https://doi.org/10.15126/surreydata.901429. Supplementary information is available. See DOI: https://doi.org/10.1039/d5na00469a.
