# Supplementary figures and images for: Exsolution of Fe-based pyramidal nanostructures from a noble metal doped perovskite matrix
Source: Nanoscale Adv. 2025 Aug 29;7(20):6426–37. doi: 10.1039/d5na00469a (PMC12424076; doi:10.1039/d5na00469a)

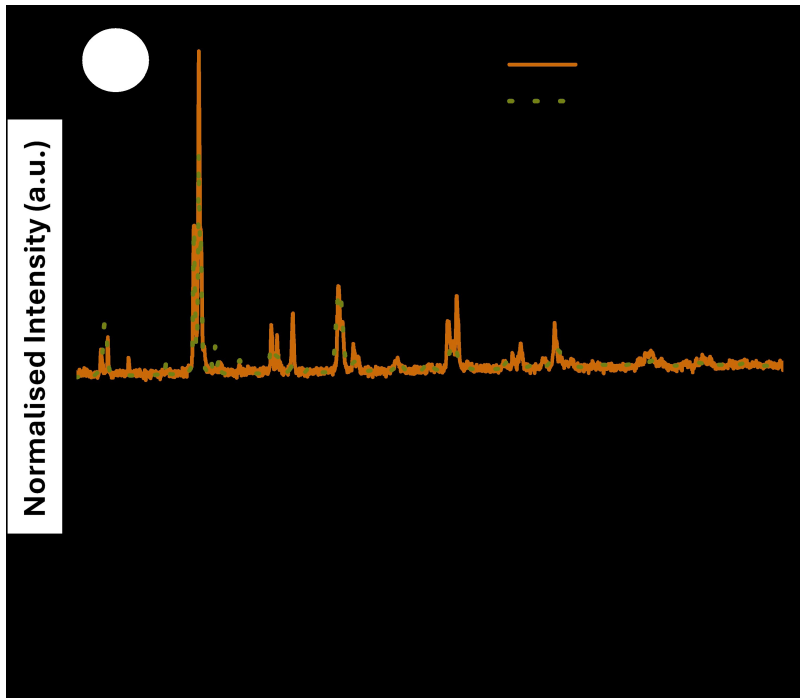

Supplement: NA-007-D5NA00469A-s002 [file NA-007-D5NA00469A-s002.pdf]

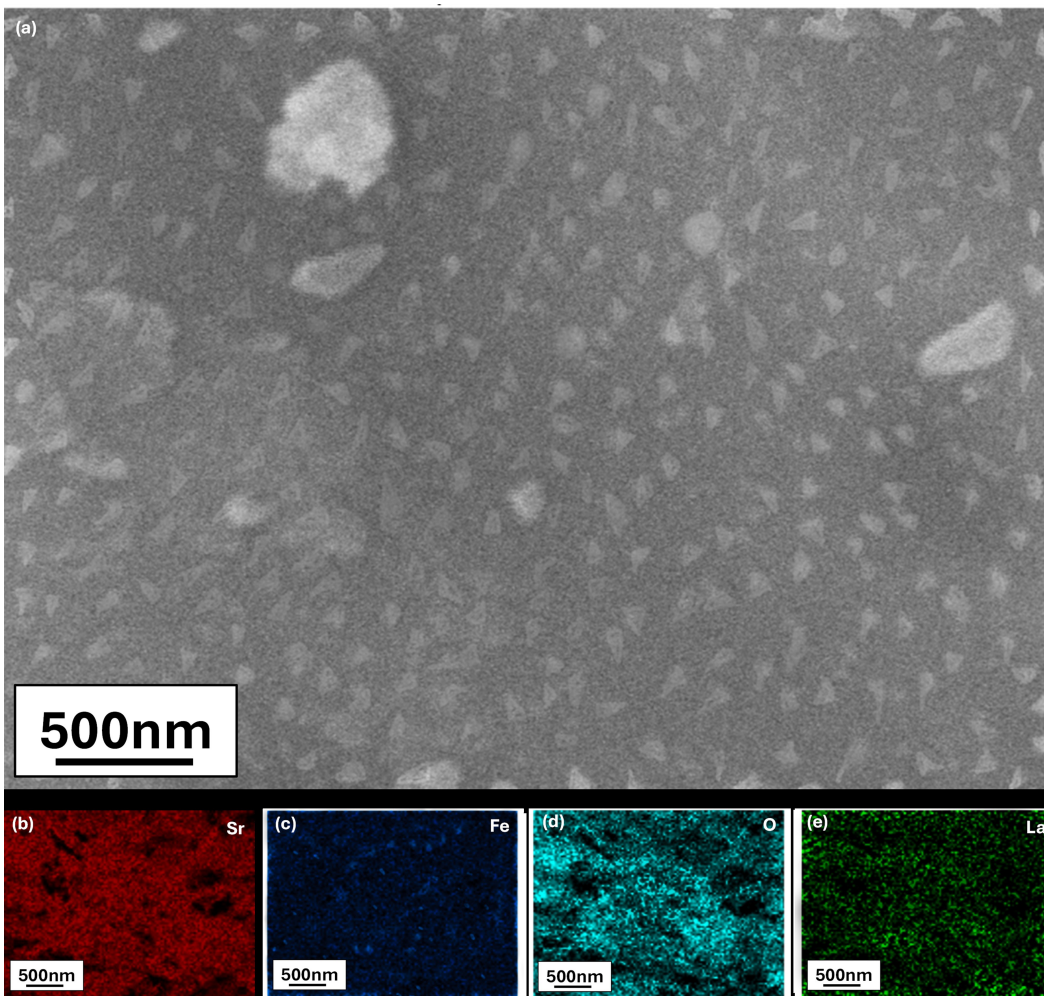

Supplement: NA-007-D5NA00469A-s003 [file NA-007-D5NA00469A-s003.pdf]

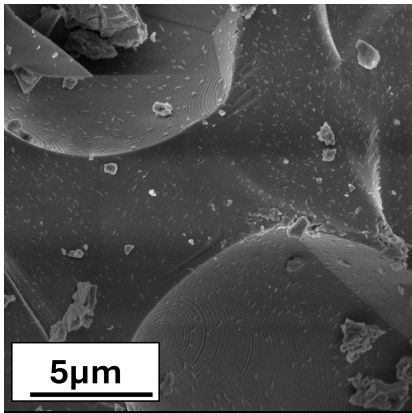

Supplement: NA-007-D5NA00469A-s004 [file NA-007-D5NA00469A-s004.pdf]

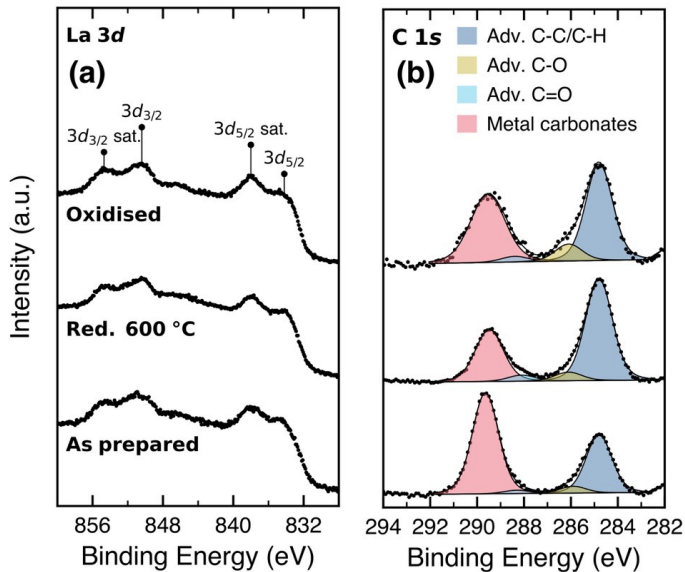

Supplement: NA-007-D5NA00469A-s005 [file NA-007-D5NA00469A-s005.pdf]

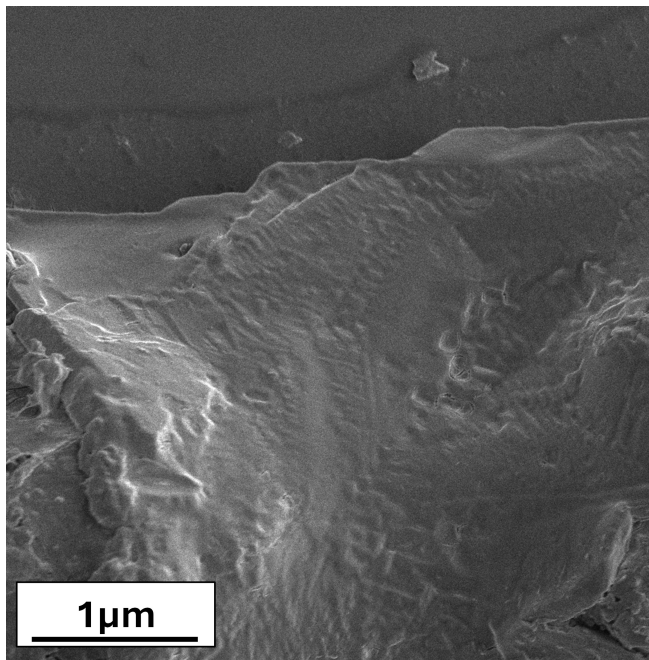

Supplement: NA-007-D5NA00469A-s006 [file NA-007-D5NA00469A-s006.pdf]

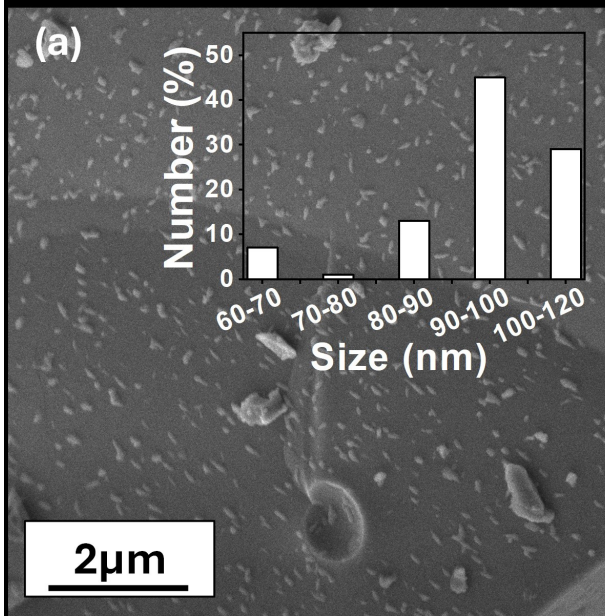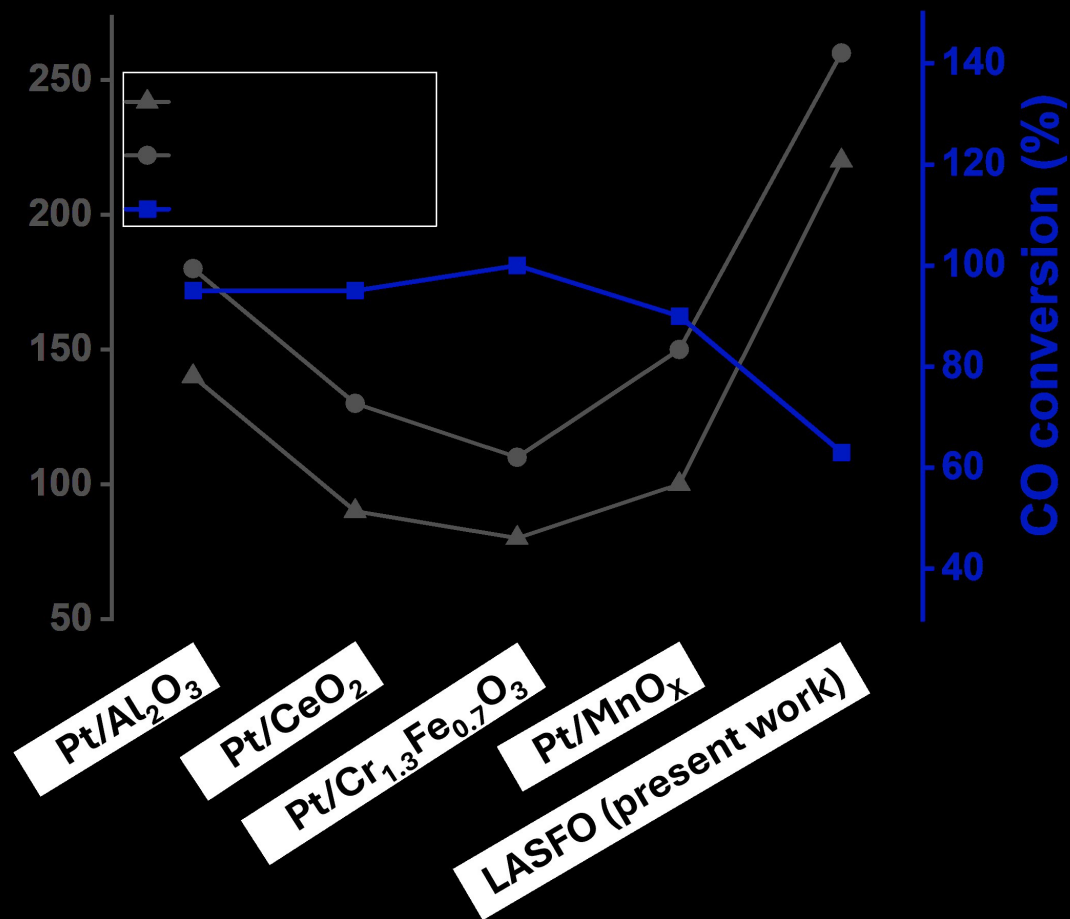

Supplement: NA-007-D5NA00469A-s007 [file NA-007-D5NA00469A-s007.pdf]
