# Supplementary material for: Exsolution of Fe-based pyramidal nanostructures from a noble metal doped perovskite matrix
Source: Nanoscale Adv. 2025 Aug 29;7(20):6426–37. doi: 10.1039/d5na00469a (PMC12424076; doi:10.1039/d5na00469a)
Supplement: NA-007-D5NA00469A-s008 [file NA-007-D5NA00469A-s008.pdf]

Supplementary Information (SI) for Nanoscale Advances.
This journal is © The Royal Society of Chemistry 2025

Supplementary Information

Exsolution of Fe-based Pyramidal Nanostructures from a Noble Metal-Doped Perovskite Matrix

Deblina Majumder[a*], Shailza Saini[b], William S. J. Skinner[c], Alex Martinez Martin[b], Gwilherm Kerherve[c], David J. Payne[c,d] , Debayan Mondal[e], Evangelos I. Papaioannou[a*], Kalliopi Kousi[b*]

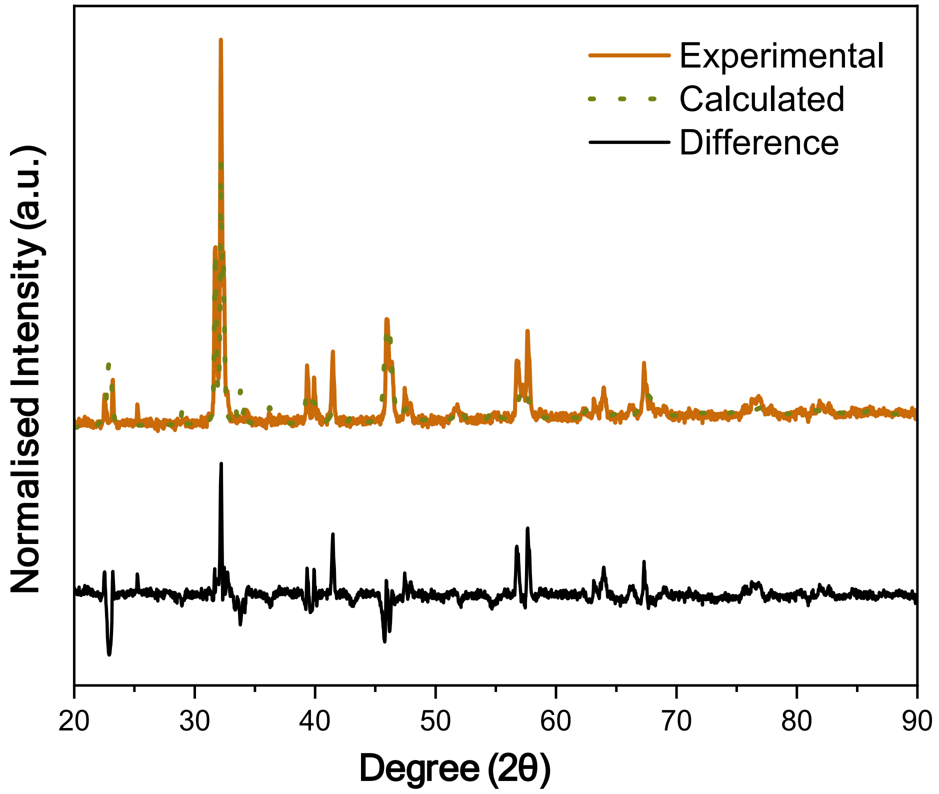

Figure S1. Refinement of tetragonal (P4/mmm) LASFO.

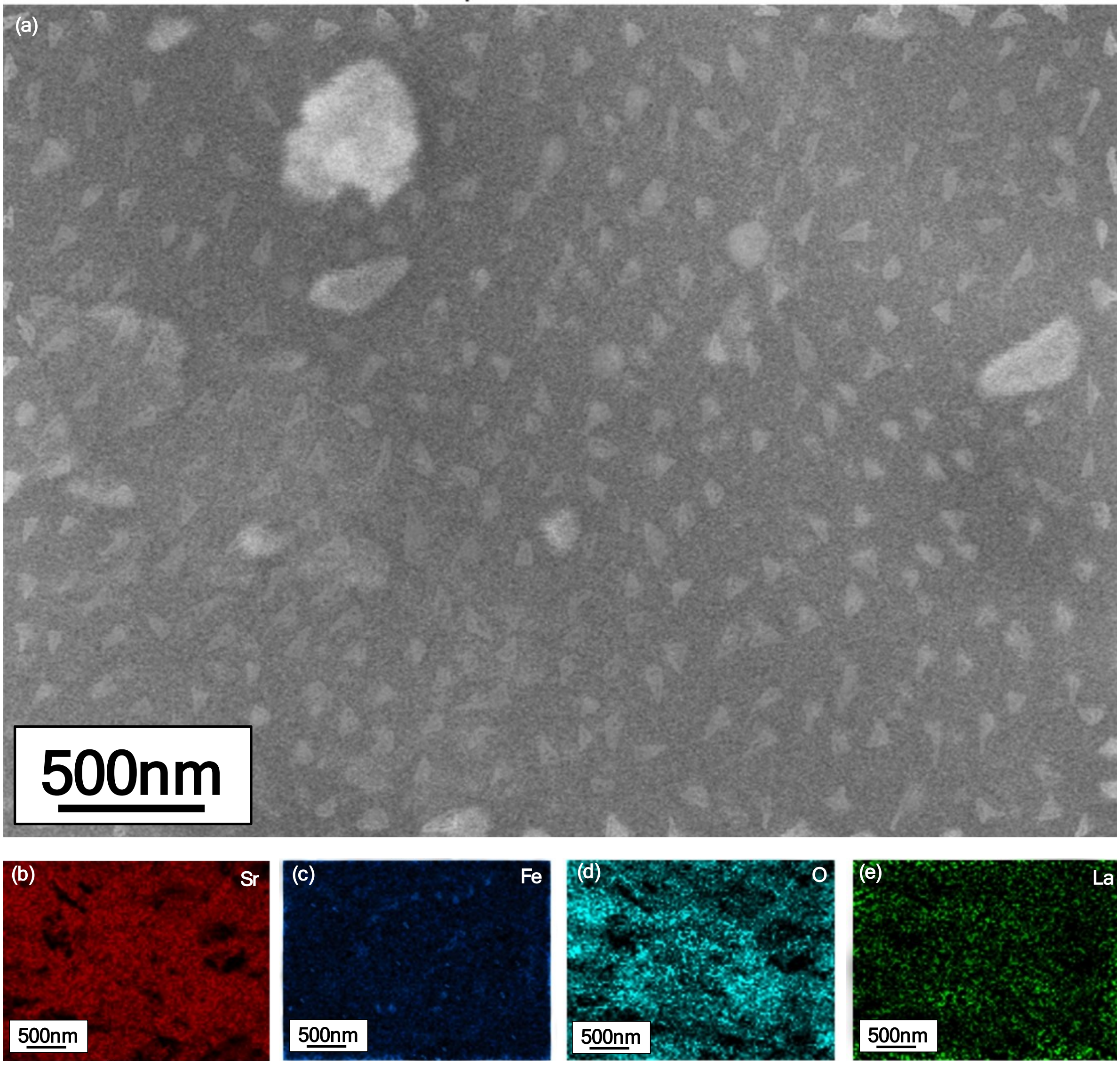

Figure S2. EDS (SEM) analysis of LASFO under low voltage. (a) represents the area of scanning and (b)-(d) shows the spatial distribution of Sr, Fe, O and La respectively.

Elements	La	Ag	Sr	Fe
Concentration (ax Ave ppm)	4.31	3.07	69.85	47.11

Table S1. ICP-OES analysis of LASFO vis axial view.

We detected La, Ag, Sr, and with final values presented in Table S1. Notably, the purpose of this analysis focused on elemental detection. Therefore, optimising for absolute accuracy was beyond the present scope. In this ICP-OES analysis, the results for La, Ag, Sr, and Fe are reported in terms average of axial view concentrations (ax Ave ppm), indicating that measurements were taken by detecting light directly along the axis of the plasma channel. This axial view configuration offers higher sensitivity and lower detection limits, making it particularly advantageous for quantifying trace elements such as La and Ag in LASFO ($La_{0.05}Ag_{0.05}Sr_{0.90}FeO_3$) sample. By capturing more of the emitted light, the axial view

enhances signal intensity, allowing for finer detection of lower-concentration elements overall. However, this increased sensitivity comes with trade-offs, as the axial view is more susceptible to interferences, particularly in complex, multi-element matrices. These interferences can arise from overlapping emission lines or matrix effects that might slightly skew results. The radial view, in contrast, detects light perpendicularly to the plasma channel, providing a higher linear dynamic range and greater stability, especially in high-matrix samples, but with lower sensitivity. Although radial view settings could mitigate some interference, axial view was chosen here to maximise sensitivity, thus ensuring reliable quantification even at trace levels for La and Ag.

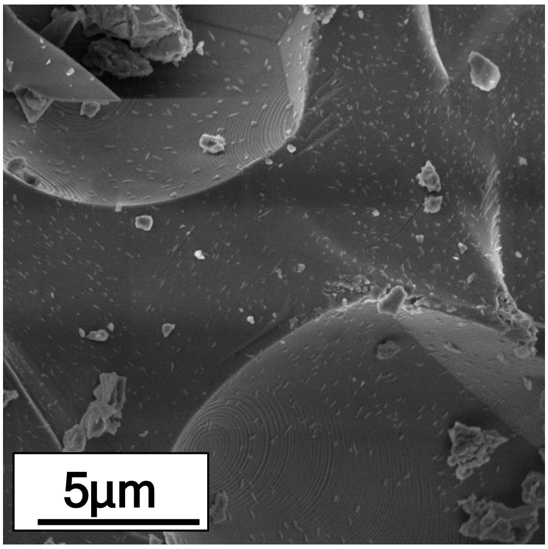

Figure S3. SEM image of post oxidation reduced LASFO.

Table S2. Quantification table for LASFO, as prepared. Relative atomic percentages are calculated in the Thermo Avantage software using TPP-2M-corrected areas (R.S.Fs provided by Thermo Scientific).

**Core level**	**Chemical state**	**Binding energy (eV)**	**FWHM (eV)**	**Raw area**	**Area (TPP-2M)**	**R.S.F**	**Rel. at. %**
Fe 2*p*	$Fe^{3/4+}$ oxide lattice	-	-	51874.79	70.96	14.353	12.45
	Fe metal	-	-	-	-	14.353	0.00
O 1*s*	LASFO lattice	528.12	1.14	7728.43	44.69	2.881	7.83
	$Fe_2O_3/Sr_2Fe_2O_5$ lattice	529.02	1.24	5057.06	29.27	2.881	5.13
	Surface hydroxyls	529.86	1.31	7172.04	41.53	2.881	7.28
	Metal carbonates	531.41	1.70	35712.51	207.06	2.881	36.27
	Adsorbed H_2O	532.92	1.74	916.51	5.32	2.881	0.93
Sr 3*d*	LASFO lattice	131.61	1.10	6409.21	19.28	4.249	3.38
	$Sr_2Fe_2O_5$ lattice	-	-	-	-	4.249	0.00
	$SrCO_3$	133.37	1.51	19751.4	59.46	4.249	10.42
C 1*s*	Adventitious C-C, C-H	284.80	1.30	2250.23	31.55	1	5.53
	Adventitious C-O	285.90	1.30	264.47	3.71	1	0.65
	Adventitious C=O	288.30	1.32	128.76	1.81	1	0.32
	Metal carbonates	289.64	1.70	3993.22	56.16	1	9.84

Table S3. Quantification table for LASFO, post-reduction. Relative atomic percentages are calculated in the Thermo Avantage software using TPP-2M-corrected areas (R.S.Fs provided by Thermo Scientific).

**Core level**	**Chemical state**	**Binding energy (eV)**	**FWHM (eV)**	**Raw area**	**Area (TPP-2M)**	**R.S.F**	**Rel. at. %**
Fe 2*p*	$Fe^{3/4+}$ oxide lattice	-	-	80648.55	110.31	14.353	18.58
	Fe metal	-	-	1333.61	1.82	14.353	0.31
O 1*s*	LASFO lattice	528.18	1.17	3669.86	21.22	2.881	3.57
	$Fe_2O_3/Sr_2Fe_2O_5$ lattice	529.01	1.47	21940.23	126.97	2.881	21.37
	Surface hydroxyls	529.78	1.23	5687.26	32.93	2.881	5.54

	Metal carbonates	531.26	1.77	22189.88	128.64	2.881	21.65
	Adsorbed H_2O	532.78	1.77	1116.94	6.48	2.881	1.09
Sr 3*d*	LASFO lattice	131.50	1.10	1739.62	5.23	4.249	0.88
	$Sr_2Fe_2O_5$ lattice	132.10	1.22	7468.43	22.47	4.249	3.78
	$SrCO_3$	133.27	1.70	13025.91	39.21	4.249	6.6
C 1*s*	Adventitious C-C, C-H	284.8	1.36	4205.44	58.96	1	9.92
	Adventitious C-O	286.1	1.36	357.49	5.02	1	0.84
	Adventitious C=O	288.1	1.36	249	3.5	1	0.59
	Metal carbonates	289.48	1.41	2231.56	31.38	1	5.28

Table S4**.** Quantification table for LASFO, after oxidation. Relative atomic percentages are calculated in the Thermo Avantage software using TPP-2M-corrected areas (R.S.Fs provided by Thermo Scientific).

**Core level**	**Chemical state**	**Binding energy (eV)**	**FWHM (eV)**	**Raw area**	**Area (TPP-2M)**	**R.S.F**	**Rel. at. %**
Fe 2*p*	$Fe^{3/4+}$ oxide lattice	-	-	65449	89.56	14.353	19.45
	Fe metal	-	-	-	-	14.353	0.00
O 1*s*	LASFO lattice	528.17	1.13	12716.58	73.54	2.881	15.97
	$Fe_2O_3/Sr_2Fe_2O_5$ lattice	529.07	1.33	5623	32.54	2.881	7.07
	Surface hydroxyls	529.96	1.47	8553.29	49.53	2.881	10.76
	Metal carbonates	531.40	1.83	15851.3	91.9	2.881	19.96
	Adsorbed H_2O	532.87	1.73	1610.14	9.35	2.881	2.03
Sr 3*d*	LASFO lattice	131.57	1.10	8788.19	26.43	4.249	5.74
	$Sr_2Fe_2O_5$ lattice	132.17	1.36	2781.86	8.37	4.249	1.82
	$SrCO_3$	133.57	1.70	8085.88	24.35	4.249	5.29
C 1*s*	Adventitious C-C, C-H	284.8	1.36	1821.49	25.54	1	5.55
	Adventitious C-O	286.1	1.36	319.26	4.48	1	0.97
	Adventitious C=O	288.4	1.36	106.27	1.49	1	0.32

	Metal carbonates	289.54	1.76	1659.7	23.34	1	5.07

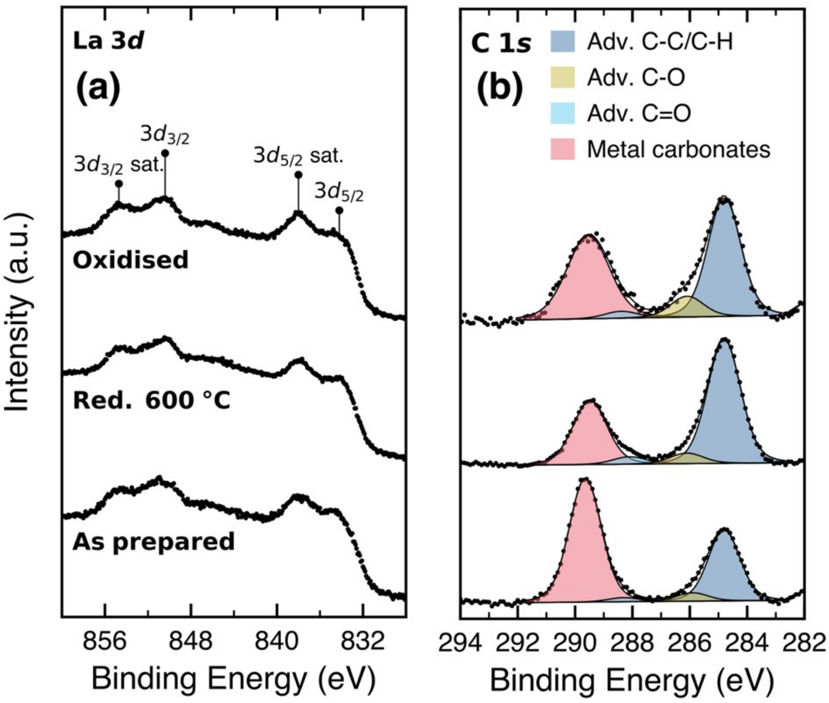

Figure S4. High-resolution core level scans of (a) La 3*d* and (b) C 1*s*. Measurements were performed on LASFO as prepared, after reduction (600 °C, 2 hours) and following subsequent oxidation. Core level spectra are normalised to the highest intensity in their spectrum to facilitate comparison between sample treatments.

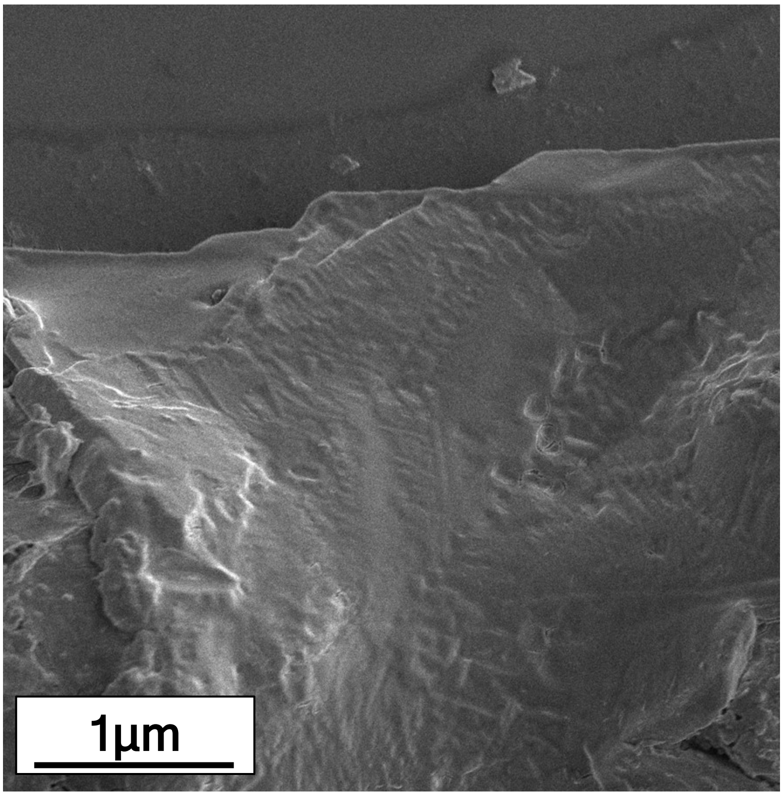

Figure S5. SEM image of LSFO without Ag doping.

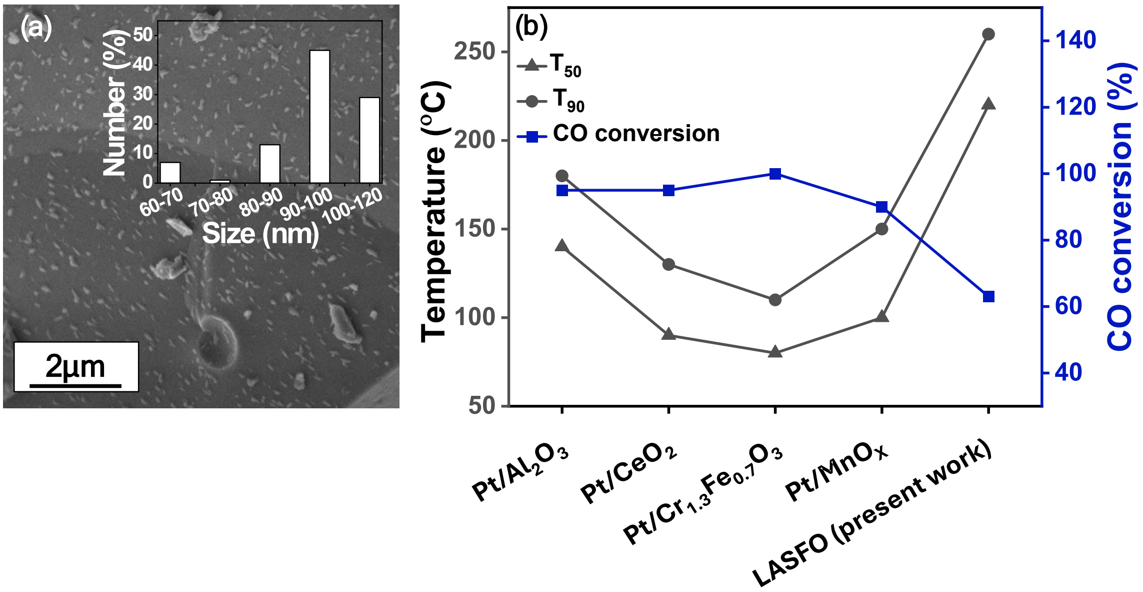

Figure S6. (a) SEM image of LASFO after cyclic redox tests with inset size distribution; (b) Comparative performance analysis plot for LASFO against traditional Pt-based catalysts such as Pt/Al_2O_3[79], Pt/CeO_2[79], $Pt/Cr_{1.3}Fe_{0.7}O_3$[78] and Pt/MnO_x[77] in CO.
